# Prevalence, Risk Factors, Prognosis, and Management of Pericardial Effusion in COVID-19

**DOI:** 10.3390/jcdd10090368

**Published:** 2023-08-27

**Authors:** İbrahim Saraç, Sidar Şiyar Aydın, Murat Özmen, Halil İbrahim Doru, Gökhan Tonkaz, Melike Nur Çırçır, Furkan Akpınar, Onur Zengin, Orhan Delice, Faruk Aydınyılmaz

**Affiliations:** 1 Department of Cardiology, Erzurum City Hospital, Erzurum 25010, Turkey; s.siyaraydin@gmail.com (S.Ş.A.); drmuratt1987@gmail.com (M.Ö.); faruk_aydinyilmaz@hotmail.com (F.A.); 2 Department of Emergency Medicine, Erzurum City Hospital, Erzurum 25010, Turkey; drhalildoru@gmail.com (H.İ.D.); melikenurcircir1996@gmail.com (M.N.Ç.); onurzengin25@gmail.com (O.Z.); orhandelice@gmail.com (O.D.); 3 Department of Radiology, Giresun University Research Hospital, Giresun 28200, Turkey; gokhantonkaz@gmail.com

**Keywords:** pericardial effusion, prevalence, prognosis, management, COVID-19

## Abstract

**Background:** There is limited data in the literature about the clinical importance and prognosis of pericardial effusion (PE) in patients discharged after recovering from COVID-19, but large-scale studies have yet to be available. This study investigated the prevalence, risk factors, prognosis, late clinical outcomes, and management of PE in COVID-19. **Materials and Methods:** Between August 2020 and March 2021, 15,689 patients were followed up in our pandemic hospital due to COVID-19. Patients with positive polymerase chain reaction (PCR) test results and PE associated with COVID-19 in computed tomography (CT) were included in the study. The patients were divided into three groups according to PE size (mild, moderate, and large). Transthoracic echocardiography (TTE) records, laboratory data, clinical outcomes, and medical treatments of patients discharged from the hospital were retrospectively reviewed. **Results:** According to the PE size (mild, moderate, large) of 256 patients with PE at admission or discharge, the mean age was 62.17 ± 16.34, 69.12 ± 12.52, and 72.44 ± 15.26, respectively. The mean follow-up period of the patients was 25.2 ± 5.12 months. Of the patients in the study population, 53.5% were in the mild group, 30.4% in the moderate group, and 16.1% in the large group. PE became chronic in a total of 178 (69.6%) patients at the end of the mean three months, and chronicity increased as PE size increased. Despite the different anti-inflammatory treatments for PE in the acute phase, similar chronicity was observed. In addition, as the PE size increased, the patients’ frequency of hospitalization, complications, and mortality rates showed statistical significance between the groups. **Conclusions:** The clinical prognosis of patients presenting with PE was quite poor; as PE in size increased, cardiac and noncardiac events and mortality rates were significantly higher. Patients with large PE associated with COVID-19 at discharge should be monitored at close intervals due to the chronicity of PE and the increased risk of tamponade.

## 1. Introduction

The coronavirus disease 2019 (COVID-19) pandemic, caused by severe acute respiratory syndrome coronavirus-2 (SARS-CoV-2), affected millions and killed hundreds of thousands of people [1]. As a result of the measures taken against the spread of the disease or reducing the effectiveness of the pathogen in the body, the severity of the disease has decreased relatively. However, COVID-19 still exists with different mutants [2]. Although COVID-19 can present mild symptoms, it can manifest with severe clinical findings such as pneumonia and acute respiratory distress syndrome (ARDS). While some patients may apply to health institutions with atypical complaints related to gastrointestinal, genitourinary, cardiovascular (CV), and other systems, others survive this process asymptomatically [3]. Additional comorbidities and demographic characteristics are essential in the course of the disease with different clinical scenarios. The acute damage and complications of the disease are well-defined. One of them is pericardial effusion (PE), which is among the pericardial syndromes [4]. It was shown in the literature that PE, which occurs in the acute period of COVID-19, is closely related to the patient’s comorbidities, the severity of lung involvement, and the history of CV disease. In-hospital mortality and intensive care hospitalization rates are quite high in COVID-19 patients accompanied by PE [5]. However, there is limited literature data regarding the clinical follow-up and prognosis of PE in patients who recovered and were discharged after this severe infection [6]. Although there are recommendations for the follow-up and treatment of idiopathic or chronic PE in the pericardial diseases guideline published in 2015 by the European Society of Cardiology (ESC) and in some publications in the literature, there are no large-scale studies for the prognosis and clinical importance of PE as a result of COVID-19 [7]. This study aimed to investigate the prevalence, risk factors, late clinical outcomes, and the effect of anti-inflammatory treatment on PE after discharge of hospitalized patients due to COVID-19.

## 2. Materials and Methods

### 2.1. Study Population

**Figure 1:**
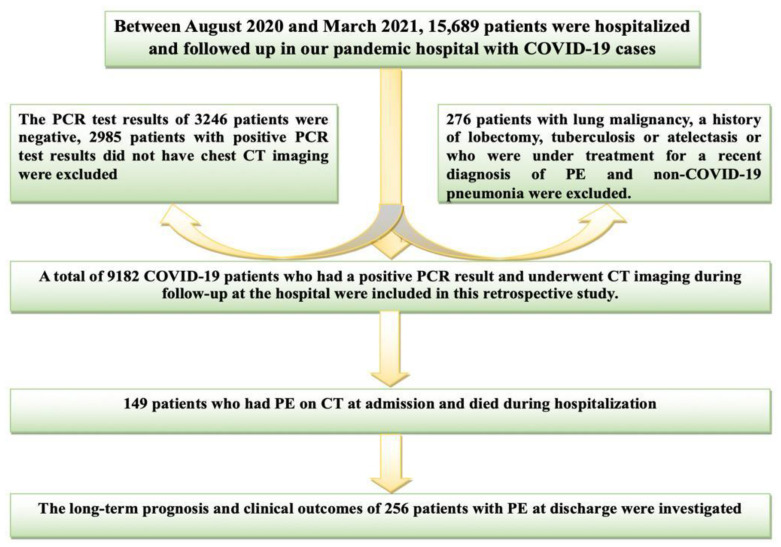
The inclusion and exclusion criteria and the flow diagram of the study.

Polymerase chain reaction (PCR) tests were routinely performed to diagnose COVID-19 in all patients. A combined swab sample was taken under the specified procedures in all patients admitted to the emergency department [8]. The patients were managed in accordance with the guidelines. These guidelines refer to large-scale, comprehensive studies [9]. Between August 2020 and March 2021, 15,689 patients were followed up in our pandemic hospital due to COVID-19. The PCR test results of 3246 patients were negative. 2985 patients with positive PCR test results did not have chest CT imaging. An additional 276 patients with lung malignancy, a history of lobectomy, tuberculosis, atelectasis, or under treatment for a recent diagnosis of PE and non-COVID-19 pneumonia were excluded. Thus, 9182 COVID-19 patients with positive PCR test results, and CT imaging at admission were included in this retrospective study. As a result of CT scanning, 405 patients with PE accompanying COVID-19 were detected. Of this patient group, 149 patients died during hospitalization. Transthoracic echocardiography (TTE) records, clinical outcomes, and medical treatments of 256 patients who were discharged after recovery were retrospectively reviewed. Laboratory samples were collected within the first 24 h after hospital admission. In addition, the patients’ CHA_2_DS_2_-VASc (C; Congestive heart failure (or Left ventricular systolic dysfunction); H, Hypertension; A_2_, Age ≥ 75 years; D, Diabetes Mellitus; S_2_, Prior Stroke or TIA or thromboembolism; V, Vascular disease (e.g., peripheral artery disease, myocardial infarction, aortic plaque); A, Age 65–74 years; Sc, Sex category (i.e., female sex); and the total severity score (TSS) scores were calculated. The study was performed in accordance with the Helsinki Declaration and with the approval of the local ethics committee. The inclusion and exclusion criteria and the flowchart of the study are shown in Figure 1.

### 2.2. The Treatment and Management of the Patient

The patients were treated according to the treatment guidelines published by the Ministry of Health. All patients were given favipiravir 2 × 1600 mg loading doses followed by 2 × 600 mg maintenance doses for 5–10 days. Patients with oxygen desaturation and lung involvement were given 6 mg/day IV dexamethasone or equivalent 40 mg/day prednisone or 32 mg methylprednisolone for 5–10 days. Patients who developed ARDS were given 250 mg/day methylprednisolone or pulse steroids (1000 mg prednisolone) for three days. Subsequently, 6 mg/day dexamethasone or 0.5–1 mg/kg/day prednisolone was given as maintenance. Inpatients who did not respond to this treatment, inpatients with macrophage activation syndrome (MAS), or patients with findings of rapidly progressive MAS, received monoclonal antibodies (MABs) via 4–8 mg/kg IV infusion, or a 400 mg standard IV single or two doses within 12 h, not exceeding a maximum of 800 mg. Appropriate empirical antimicrobial therapy (beta-lactam, macrolide, and quinolone) was initiated if clinical imaging or microbiological examination showed signs of sepsis or findings suggestive of secondary bacterial infection. Prophylactic low molecular weight heparin was given to patients without contraindications [10,11]. Patients diagnosed with pericarditis or PE due to COVID-19 were followed up with the treatment recommendations in the literature; in all three groups, colchium or NSAIDs treatment was started within patients who were evaluated as pericarditis in the primary diagnosis in the etiology of PE. Steroids were combined with treatment in some patients. PE was attributed to an inflammatory process other than pericarditis in patients with severe lung involvement and hypoxia. In these patients, only steroid treatment was prioritized [12].

The usual dosing for ibuprofen is 600 mg every eight hours for 1–2 weeks in suspected cases of pericarditis; treatment duration is guided by symptoms and C-reactive protein (CRP), but generally 1–2 weeks for uncomplicated cases. For colchicine, the recommended dose is 0.5 mg twice daily, whereas dose adjustments should be performed taking into account age and body weight (it is advised to consider halving the dose in patients aged >70 years and in those weighing <70 kg) as well as creatinine clearance [7,12].

### 2.3. Pericardial Effusion (CT and TTE)

The diagnosis of pericardial effusion is generally performed via echocardiography, which also enables semiquantitative assessment of the pericardial effusion size and its hemodynamic effects. Although echocardiography remains the primary diagnostic tool for the study of pericardial diseases because of its widespread availability, portability, and limited costs, CT and CMR provide a larger field of view, allowing the detection of loculated pericardial effusion and pericardial thickening and masses, as well as associated chest abnormalities [7]. At the beginning of the COVID-19 pandemic, patients were not administered routine TTE for the etiology of dyspnea, and a significant proportion of inpatients did not have TTE (within the framework of the isolation rules taken due to the high contagiousness of the disease) [13]. Therefore, a retrospective evaluation of PE was made with the findings in CT. The smallest amount of pericardial fluid detectable by CT is approximately 10 mL [14]. The presence of >4 mm fluid between both pericardial layers on CT is considered abnormal. In this study, the classification of PE size in CT was performed as in the classification model according to TTE (mild < 1 cm, moderate 1–2 cm, large > 2 cm) [7].The evaluation and classification of PE at the mean 3rd month, mean 12th month, and long term in TTEs were performed at the post-discharge controls; apical 4-chamber, parasternal long axis, parasternal short axis, and subcostal imaging were evaluated by measuring the widest length distance in diastole [7,15].

### 2.4. Pulmonary Involvement and Total Severity Score

Semiquantitative visual severity scores of lung involvement of patients who had PE on CT at admission and were discharged after recovery was calculated. This method is an adaptation of a method previously used to describe CT findings that correlated with clinical and laboratory parameters in COVID-19 patients, and the percentage of involvement of each five lung lobes was calculated semi-quantitatively, i.e., visually. Two radiologists, blinded to the clinical data, evaluated the CT findings in consensus. Total severity score (TSS, potential values from 0 to 20) was computed by summing up individual scores from 5 lung lobes; scores of 0, 1, 2, 3, or 4 were assigned, respectively, for each region if parenchymal opacification involved 0%, 1–25%, 25–50%, ≥50–75%, or 75–100% of that region [16].

### 2.5. Chest CT Scan

All CT images of lung parenchyma were reviewed at a window width and level of 1000 to 2000 Hounsfield units (HU) and −700 to −500 HU, respectively. Chest CT imaging was performed using a Toshiba Aquilion 64-detector CT scanner (Otawara, Japan). All patients were examined in a supine position, and CT images were acquired during a single inspiratory breath hold. The scanning range was from the apex of the lung to the costophrenic angle. CT scan parameters were X-ray tube parameters 120 kVp, 110–270 mAs, anfFoV 400 mm; section thickness 5 mm.

### 2.6. Statistical Analysis

Analyses were made with the IBM SPSS 20 statistical analysis program. Data were presented as mean, standard deviation, median, minimum, maximum, percentage, and number. The normal distribution of continuous variables was evaluated with the Shapiro–Wilk-W test, Kolmogorov–Smirnov test, Q-Q plot, skewness, and kurtosis. In comparing continuous variables with more than two independent groups, the ANOVA test was used when the normal distribution condition was met, and the Kruskal–Wallis test was used when it was not. Post hoc tests after the ANOVA test were performed using the Tukey test when variances were homogeneous, and Tamhane’s T2 test when variances were not homogeneous. After the Kruskal–Wallis test, posthoc tests were performed using Kruskal–Wallis 1-way ANOVA (k samples) test—2 × 2 between categorical variables. In comparisons, the expected value (>5) was performed using the Pearson Chi-square test; if the expected value was between (3–5), the chi-square Yates test was used; and if the expected value was (<3), the Fisher’s-Exact test was used. For comparisons greater than 2 × 2 between categorical variables, the Pearson Chi-square test was used in the case of the expected value (>5), and the Fisher-Freeman-Halton test was used in the case of the expected value (<5). The statistical significance level was accepted as *p* < 0.05.

## 3. Result

Pericardial effusion was observed in 405 (4.41%) patients out of 9182 with lung involvement and PCR (+). Of the patients with PE, 149 (39.2%) died during hospital follow-up. The data of the remaining 256 (63.2%) patients were analyzed. According to the PE size (mild, moderate, and large), the mean age was 62.17 ± 16.34, 69.12 ± 12.52, and 72.44 ± 15.26, respectively. The mean follow-up period was 25.2 ± 5.12 months. The correlation between the severity of lung involvement and PE size, which tended to increase with age, was statistically significant in all three groups. Inflammatory and metabolic parameters of the groups showed statistically significant as lung involvement and PE size increased. In addition, as the PE size increased in the posthoc analysis, statistical significance was observed in the TSS and CHA_2_DS_2_-VAS_C_ scores, CRP, duration of hospitalization, SO_2_, and lymphocyte among all groups (Table 1).

Patients with a large PE had higher rates of comorbid diseases. The use of chronic cardiovascular drugs was higher in patients with large PE. As the PE size increased, the need for intensive care, monoclonal antibodies (MABs), and pulse steroid requirements increased and showed statistical significance (Table 2).

Table 3 shows the change in PE at an average of 3 months, according to the nonsteroid anti-inflammatory drugs (NSAIDs), steroid, and colchicine treatment taken by the patients who recovered and were discharged. Although the change in PE in the 3rd month on average, according to the treatment received, was clinically different in all three groups, it did not show statistical significance. PE was observed in 11 patients without pulmonary involvement (TSS:0), five patients received colchicine only, three patients received NSAIDs only, and three patients received only steroids. At the end of the mean three months, PE became chronic in 178 (69.6%) patients, and chronicity was observed in 70 (51%) patients in the mild group. In addition, a similar chronicity rate was observed in the in-group comparison in all treatment groups (Table 3).

Although the cardiac origin late clinical outcomes were more common in the group with large PE, no statistical significance was observed. However, the frequency of hospitalization for noncardiac reasons was statistically significant in patients with a large PE size. While the rates of at least two vaccination doses were similar in all three groups, the rate of the inactivated virus was higher in the group with a large PE size. Cardiac tamponade developed in 1 (0.07%) patient in the mild group, 3 (3.84%) patients in the moderate group, and 5 (12.19%) patients in the large group during the total follow-up period. During the follow-up of the patients, symptoms such as palpitation, dyspnea, and chest pain were questioned. No asymptomatic patients were in the large PE group; dyspnea was the most common symptom (74%). 33% of the mild group remained asymptomatic in their follow-up. The total mortality and death from cardiac or noncardiac etiologic causes were statistically significant between the groups. (*p*: 0.001) The risk of all-cause mortality increased as the PE size increased. Vaccination rates were lower in patients who died (Table 4).

**Figure 2:**
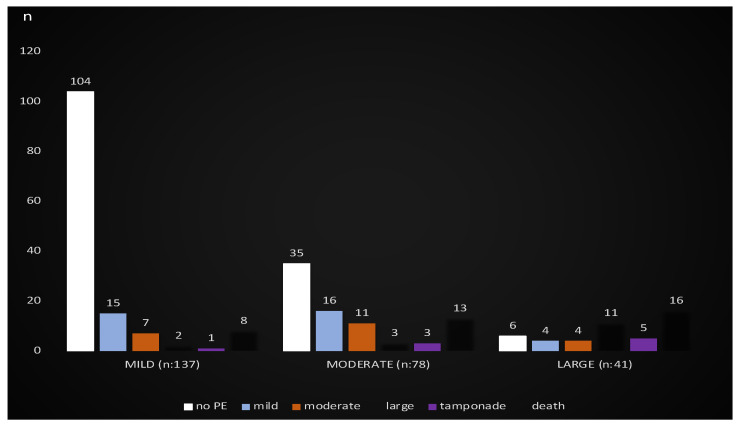
PE change after a mean follow-up of 25 months.

After a mean follow-up of 25 months, PE was not observed in 104 (75.9%) patients in the initial mild group. PE was not observed in 35 (44.8%) patients in the moderate group. Again, in the group with large PE in 41 patients at the beginning, PE was not observed in 6 (14.6%) patients in the late period. The numbers of PE dimensions at baseline and mean follow-up 25 months are shown in Figure 2. It was observed that one patient in the large PE group developed chronic effusive-constrictive pericarditis (CP) in the controls at the 6th-month follow-up. The patient, who initially had mild symptoms and signs (cardiac filling abnormalities and diastolic heart failure), was given high-dose anti-inflammatory therapy (colchicine and NSAIDs-Ibuprofen) and diuretic therapy for peripheral congestion. During the 2-week follow-up, pericardiectomy was performed because the patient’s clinical condition worsened and there was no response to treatment. In addition, cardiac tamponade was observed in 8 patients during the follow-up period. (Figure 2) Successful pericardiocentesis was performed in 7 patients. In 1 patient, drainage was tried but was unsuccessful, and so was referred to surgery. Among these patients who developed cardiac tamponade and underwent pericardiocentesis, 1 patient, who was initially in the moderate PE group and diagnosed with COPD and AF, died due to respiratory failure at the next hospitalization. The patient, who was initially in the large PE group and underwent surgical pericardiocentesis, was diagnosed with HT, DM, and CKD, and died in the hospital due to multi-organ failure after the procedure. One of the 2 patients in the large PE group, who developed tamponade and underwent pericardiocentesis, had reduced ejection fraction heart failure and additional comorbidities, and died as a result of out-of-hospital arrest. The other patient died while being followed in the hospital diagnosis from decompensated heart failure. The follow-up of the other 4 patients who underwent pericardiocentesis continues, and complete regression was achieved in 2 patients. The other 2 have mild PE.

## 4. Discussion

In our study, it was determined that the presence of PE is a poor prognosis factor in the early and late periods in patients hospitalized for COVID-19 and follow-up periods. In addition, as the PE size of the patients increased, adverse cardiovascular outcomes increased in the early and late periods.

Although we know enough about the acute cardiac effects and complications of COVID-19, we still need more data on chronic cardiac manifestations [17]. One of these chronic cardiovascular pathologies is PE. The pathophysiology of PE seen during the COVID-19 process has been tried to be explained in various ways: (a) Direct invasion of cardiomyocytes with a virus (binding to ACE2 receptor) results in myocardial injury, myocarditis, and cardiomyopathy; (b) Pericarditis or myopericarditis occurs with indirect effects of inflammatory cytokines (TNF-a, IL-1, IL-6); (c) As a result of ARDS or hypoxia triggering a myocardial injury or pulmonary hypertension; (d) Finally, it was suggested that PE occurs as a result of direct involvement of the pericardium [4]. Hypotheses that PE associated with COVID-19 arose during a severe inflammatory process rather than direct pericardial involvement are at the forefront [18]. As a result of these processes, although PE may appear alone, it can also accompany ARDS or lung involvement, cardiomyopathy, pericarditis, and myopericarditis [4]. In these clinical scenarios, dynamic changes in many inflammatory markers, enzymes and biomarkers are observed in the serum. One of them is troponin. Troponin is an important marker in showing cardiac involvement caused by primary and secondary causes. However, indicating that when used alone, troponin may not be a useful predictor of primary cardiac involvement, early and late complications in COVID-19. In fact, significant troponin change was also shown to occur in only a low percentage of COVID-19 patients with cardiac abnormalities on CMR [19]. In our study, serum troponin levels were statistically significant between group 1 and other groups. However, no difference was observed between groups 2 and 3. As a result, no correlation could be established between troponin levels in predicting complications and differences in early and late clinical outcomes of group 2 and group 3 patients. This indicates the need for different biomarkers that can predict the long-term CV outcomes of COVID-19. For example, microRNAs, which are estimated to regulate the expression of more than 60% of protein-coding genes in mammals, were shown to play a key role in many physiological and pathological mechanisms, including the antiviral response. MiRNAs are involved in the regulation of complications of COVID-19, particularly acute and chronic CV events [20].

The frequency of PE in the acute period of COVID-19 was observed as 4.6% [18]. In addition, it was reported that PE is seen between 5% and 20% in the chronic period [19,21]. Similarly, in our study, the incidence of PE in the acute period was found to be 4.41%. Most of these patients (49.4%) became chronic as mild PE. Patients were divided into three groups (mild, moderate, and large) regarding PE size. PE size was also increased in patient groups with signs of COVID-19 disease severity, such as advanced age, increasing comorbid conditions, the severity of pulmonary involvement, high CHA_2_DS_2_-VAS_C_ score, hospitalization in intensive care, patients in need of MABs and pulse steroids, and high inflammatory parameters. In addition, as the PE size increased, the frequency of hospitalization for recurrent cardiac and noncardiac reasons, complications, and mortality rates increased. In studies conducted with follow-ups after COVID-19, it was shown that the risk of cardiovascular events, including pericardial diseases, increases further compared to the period before the COVID-19 pandemic. Comorbid conditions, advanced age, and gender contributed to this condition [22]. We can say that the presence of PE accompanying COVID-19 patients is a major risk factor and a poor prognostic indicator in terms of complications in patients in the acute and chronic phases.

There was no consensus in the literature on the management of PE associated with COVID-19, and patients were previously treated on a case-by-case basis. In cases other than COVID-19, colchicine is recommended to be added in the first attack or recurrent pericarditis, in addition to NSAIDs or acetylsalicylic acid (ASA) [7]. In the early part of the COVID-19 pandemic, colchicine was used to directly or indirectly reduce inflammation for virus-related pericarditis or PE [23]. Initially, using NSAIDs in COVID-19 patients was not recommended, as these agents were believed to facilitate virus invasion into cells by increasing ACE2 receptor expression. However, in the following period, it was seen that the concerns about the adverse effects of NSAID use in COVID-19 patients were unfounded, and the use of these agents was also recommended [12,24]. If the etiology is unknown in pericardial diseases, limited steroid therapy is recommended, as it is an independent risk factor for recurrence and may impair viral clearance [25]. In PE accompanying COVID-19, it was suggested that steroids can be used, especially in conditions such as severe respiratory disease or in patients unresponsive or intolerant to NSAIDs [12]. The positive effect of steroid therapy was demonstrated in patients who did not respond to conventional therapy for the pericardial syndrome that persisted after COVID-19. However, the steroid dosage and use duration in this study differ from ours [26]. It has been emphasized that post-COVID-19 pericarditis or PE may also be associated with ongoing inflammation with the persistence of viral nucleic acid without virus replication in the pericardium. Therefore, steroid therapy has been a viable option for patients who do not respond to or cannot tolerate conventional therapy and require treatment against the pericardial inflammatory process rather than an acute viral injury to the pericardial tissue [12]. In our study, the patients were divided into groups according to their steroid, colchicine + steroid, and NSAID treatment. When these groups were compared in terms of chronicity of PE or the dynamic change at an average of 3 months, the mild group showed significantly less chronicity than the other groups. However, statistical significance was not observed in comparing groups according to medical treatment. A severe inflammatory process accompanies PE associated with COVID-19. We can say that the anti-inflammatory treatment options used in our patients are appropriate [12]. In cases where CRP is normal in patients with PE, it is not recommended to administer any anti-inflammatory therapy alone or in combination, including ASA, NSAIDs, steroids, or colchicine [7]. In our study, the CRP levels of nearly all of our patients were above normal and statistically significant between the groups. However, it was not observed that the anti-inflammatory drugs used in all groups were superior to each other on chronicity and regression within the groups.

Cardiac tamponade is a life-threatening condition in the chronic process of COVID-19 as well as in the acute period [6]. In a study of 28 patients on the follow-up and prognosis of chronic PE, 30% progression in cardiac tamponade was observed in the late period, which is considered a relatively high rate [27]. PE follow-up frequency was short in these patients, and the pericardial drainage intervention threshold was kept low. However, in recent studies, the progression of chronic PE cases to tamponade has been seen at a lower rate [28]. In a study of patients with chronic, large PE, of whom 56% were symptomatic, and evaluating late outcomes, idiopathic/viral chronic large PE was generally benign, with reduced effusion size in most cases, and complete regression detected in approximately 40%. Cardiac tamponade was observed in 8% during an average follow-up of 50 months, and the risk of developing tamponade was 2.2% per year [29]. In a prospective study lasting 31 months, in which post-MI patients were excluded, the outcome of idiopathic/viral pericarditis and pericarditis of known etiology (autoimmune, neoplastic, tuberculosis, purulent, etc.) was compared. According to this, patients with known etiology had a higher complication rate (18.2% vs. 18.0%; *p* < 0.001), more cardiac tamponade (0.8% vs. 14.5%; *p* < 0.001), and more mortality had been observed [30]. In our study, tamponade developed in 1 (0.7%) in the mild group, 3 (3.84%) in the moderate group, and 5 (12.19%) in the large group. The frequency of tamponade increased as the initial PE size increased, and it was statistically significant. Compared to idiopathic PE, the frequency of tamponade and mortality rates were higher in a large group [29]. In addition, the rate of complete regression in chronic large PE was quite low compared to the literature, which can be explained by the shorter follow-up period compared to the other studies [29]. COVID. Therefore, in our study, it was observed that the frequency of PE becoming chronic in COVID-19 patients and the frequency of tamponade progression in patients with initially large PE were more common than in patients with other viral/idiopathic PE. In addition, CP was observed in one patient in our study. Although there is no clear literature information on the incidence of constrictive pericarditis after COVID-19, the incidence of CP after viral pericarditis and/or viral-induced PE is quite rare [7].

During COVID-19, vaccination has shown its effectiveness in controlling the disease [31]. In our study, the rate of those receiving at least two vaccine doses in the PE groups was similar. Again, the rate of inactivated vaccines was similar in all groups. However, the rate of mRNA-based vaccine implementation decreased as PE size and TSS score increased. The vaccination rate was lower in patient groups who died and was statistically significant. The positive effect of vaccination on mortality was already demonstrated in this process [32]. All patients in the large PE group were symptomatic at visits. Approximately 25% of patients in the mild PE group were asymptomatic. The most common symptom in these patients was dyspnea. The symptom rate was higher compared to the literature [29,33]. However, studies in the literature included non-COVID-19 chronic PE. In our study, as the causes of the symptoms, it is thought that the chronic effects of pulmonary involvement due to COVID-19 continue with pericardial involvement [34]. This situation can be shown as one of the main reasons that negatively affect the functional capacity, fragility, comorbidities, and clinical progression of PE in patients with PE accompanying COVID-19.

## 5. Limitations

Firstly, this study was designed retrospectively, and the data were obtained from files or electronic records. At the beginning of the COVID-19 pandemic, patients were not administered routine TTE for the etiology of dyspnea, and a significant proportion of inpatients did not have TTE. Therefore, the PE findings of the patients during the hospitalization period were evaluated with CT. Detailed data on myocarditis accompanying PE could not be obtained. Anti-inflammatory treatment doses and durations of the patients in the service and intensive care units differed according to the follow-up period and the severity of the disease.

## 6. Conclusions

The clinical prognosis of patients presenting with PE was quite poor in COVID-19. As PE in size increased, cardiac and noncardiac events and mortality rates were significantly higher. Although many mechanisms were proposed for the pathophysiology of PE associated with COVID-19, increased excessive inflammation is an important cause. The incidence of chronicity and recurrence was similar in patients with acute COVID-19 with PE treated with steroids, NSAIDs, and colchicine. As COVID-19-related PE increases in size and progresses to chronic PE, there may be increased cardiac and noncardiac outcomes, tamponade, and mortality rates. Patients with PE in the acute period should be followed closely due to the chronicity and the increased risk of tamponade. Clarifying the pathophysiology and specificity in treatment may affect the rates of chronicity in follow-up. For this, more comprehensive and prospective studies are needed.

## Figures and Tables

**Table 1:** Comparison of three PE groups according to age, TSS/CHA_2_DS_2_VAS_C_ score, and laboratory parameters at the time of admission to the hospital.

	PE in CT (Group): MILD:0, MODERATE:1, LARGE:2						*p* Value	Posthoc
	0		1		2			
	N	Mean ± SD	N	Mean ± SD	N	Mean ± SD		
TSS	137	6.42 ± 5.03	78	9.53 ± 4.49	41	14.63 ± 3.34	0.000	ALL
Age (years)	137	62.17 ± 16.34	78	69.12 ± 12.52	41	72.44 ± 15.26	0.000	ALL
CHA_2_DS_2_VASC_s_	137	2.12 ± 1.69	78	3.13 ± 1.72	41	4.51 ± 2.04	0.000	ALL
Duration of Hospitalizations/days	137	8.69 ± 5.084	78	13.23 ± 6.877	41	20.93 ± 16.015	0.000	ALL
Albumin (g/dL)	129	44.88 ± 5.20	72	41.30 ± 7.17	34	39.78 ± 5.51	0.000	0–1, 0–2
Creatinine (mg/dL)	115	88.17 ± 29.38	65	81.59 ± 45.37	34	74.23 ± 38.39	0.029	0–1
CRP (mg/L)	137	17.77 ± 29.18	78	45.20 ± 56.64	41	80.81 ± 80.60	0.000	ALL
Troponin I (ng/mL)	137	0.22 ± 0.39	78	1.30 ± 2.37	41	2.38 ± 7.75	0.000	0–1, 0–2
SO2 (pulse oximeter,%)	137	84.68 ± 6.14	78	81.72 ± 8.54	41	76.78 ± 8.74	0.000	ALL
Hb (g/dL)	132	14.18 ± 2.04	77	13.75 ± 2.17	36	13.14 ± 2.02	0.041	0–2
Lymphocyte count (10^3^/μL)	126	1.80 ± 1.83	77	1.08 ± 0.45	36	0.83 ± 0.29	0.000	ALL
Ferritin (ng/mL)	137	408.45 ± 583.93	78	588.67 ± 983.52	41	889.59 ± 1470.30	0.033	0–2
D-dimer (μg/mL)	137	728.96 ± 602.37	78	1810.94 ± 2269.93	39	2243.38 ± 4187.28	0.000	0–1, 0–2
Wbc (10^3^/μL)	132	7.40 ± 3.42	77	10.66 ± 5.26	37	12.89 ± 7.23	0.000	0–1, 0–2
Platelet count (10^3^/μL)	132	126.12 ± 83.06	78	118.71 ± 67.11	36	112.50 ± 94.57	0.340	
Average long-term follow-up (month)	137	25.28 ± 4.56	78	25.94 ± 4.62	41	23.49 ± 7.34		
Mean 3rd-month LVEF	128	54.87 ± 4.68	71	52.56 ± 7.34	36	50.73 ± 9.63	0.000	0–1, 0–2
Long-term mean LVEF	119	54.76 ± 5.16	59	52.19 ± 7.77	23	48.11 ± 10.43	0.000	0–1, 0–2

TSS, total severity score; CT, computerized tomography; CRP, C-reactive protein; SO_2_, oxygen saturation; Hb, hemoglobin; Wbc, white blood cell; CHA_2_DS_2_VASC: C; Congestive heart failure (or Left ventricular systolic dysfunction); H, Hypertension; A_2_, Age ≥ 75 years; D, Diabetes Mellitus; S_2_, Prior Stroke or TIA or thromboembolism; V, Vascular disease (e.g., peripheral artery disease, myocardial infarction, aortic plaque); A, Age 65–74 years; Sc, Sex category (i.e., female sex); SD, Standard Deviation; LVEF, left ventricle ejection fraction.

**Table 2:** Comparison of comorbidities, medications, and needs for intensive care, pulse steroids, and MABs during follow-up.

	PE in CT (Group): MILD:0, MODERATE:1, LARGE:2			*p* Value
	0 N: 137	1 N: 78	2 N: 41	
	N %	N %	N %	
Need for ICU, n (%)	9 (6.57%)	22 (28.21%)	32 (78.05%)	<0.001
MABs, n (%)	1 (0.73%)	1 (1.28%)	5 (12.20%)	0.002
Pulse Steroid, n (%)	6 (4.38%)	5 (6.41%)	10 (24.39%)	0.001
Gender/Male, n (%)	76 (55.47%)	45 (57.69%)	25 (60.98%)	0.817
DM, n (%)	26 (18.98%)	26 (33.33%)	21 (51.22%)	<0.001
HT, n (%)	52 (37.96%)	51 (65.38%)	28 (68.29%)	<0.001
CAD, n (%)	19 (13.87%)	22 (28.21%)	23 (56.10%)	<0.001
HF, n (%)	8 (5.84%)	14 (17.95%)	18 (43.90%)	<0.001
COPD, n (%)	21 (15.33%)	17 (21.79%)	20 (48.78%)	<0.001
CVD, n (%)	3 (2.19%)	5 (6.41%)	6 (14.63%)	0.008
AF, n (%)	16 (11.68%)	13 (16.67%)	11 (26.83%)	0.063
CRF, n (%)	11 (8.03%)	15 (19.23%)	12 (29.27%)	0.001
PAD, n (%)	8 (5.84%)	4 (5.13%)	4 (9.76%)	0.568
ASA, n (%)	20 (14.60%)	23 (29.49%)	17 (41.46%)	0.001
Statin, n (%)	13 (9.49%)	11 (14.10%)	14 (34.15%)	0.001
Clopidogrel, n (%)	4 (2.92%)	8 (10.26%)	8 (19.51%)	0.001
BB, n (%)	35 (25.55%)	32 (41.03%)	28 (68.29%)	<0.001
Furosemide, n (%)	7 (5.11%)	11 (14.10%)	12 (29.27%)	<0.001
CCB, n (%)	9 (6.57%)	16 (20.51%)	6 (14.63%)	0.007
ACEI-ARB	42 (30.66%)	40 (51.28%)	24 (58.54%)	0.001
Spirinolactone, n (%)	4 (2.94%)	8 (10.26%)	9 (21.95%)	<0.001
OAC, n (%)	9 (6.57%)	10 (12.82%)	7 (17.07%)	0.08
non-medicated, n (%)	68 (49.64%)	11 (14.10%)	6 (14.63%)	<0.001

CT, computerized tomography; ICU, intensive care unit; MABs, monoclonal antibodies; DM, diabetes mellitus; HT, hypertension; CAD, coronary artery disease; HF, heart failure; COPD, chronic obstructive pulmonary disease; CVD, cerebrovascular disease; AF, atrial fibrillation; CRF, chronic renal failure; PAD, peripheral artery disease; ASA, acetylsalicylic acid; BB, beta blocker; CCB, calcium channel blockers; ACEI, angiotensin-converting enzyme inhibitor; ARB, angiotensin receptor blocker; OAC, oral anticoagulant.

**Table 3:** Change in PE size from baseline at the end of 3rd month according to NSAIDs, colchicine, and steroid therapy status in patients evaluated as COVID-19-related PE at admission.

PE Size	Medications at Admission or Discharge			PE Size at Mean 3rd Month			*p* Value
			Mild/n	Moderate/n	Large/n	No PE/n	
Mild	NSAIDs/n	49	23	2	1	23	0.209
	Colchıum + Steroid/n	15	6	2	1	6	
	Only Steroid/n	65	20	7	0	38	
Moderate	NSAIDs/n	15	6	9	0	0	0.104
	Colchıum + Steroid/n	23	10	6	1	6	
	Only Steroid/n	40	19	18	0	3	
Large	NSAIDs/n	5	0	3	2	0	0.368
	Colchıum + Steroid/n	29	0	10	19	0	
	Only Steroid/n	7	0	4	3	0	

PE, pericardial effusion; NSAIDs, Non-steroidal anti-inflammatory drugs.

**Table 4:** Late clinical outcomes of the patients.

Hospitalization		PE in CT (Group): MILD:0, MODERATE:1, LARGE:2			*p* Value
		0	1	2	
		N %	N %	N %	
Cardiac etiology	No Hospitalization	113 (82.48%)	47 (60.26%)	8 (19.51%)	NA
	AF	10 (7.30%)	10 (12.82%)	9 (21.95%)	
	HF	5 (3.65%)	11 (14.10%)	10 (24.39%)	
	ACS	7 (5.11%)	8 (10.26%)	8 (19.51%)	
	Other	2 (1.46%)	2 (2.56%)	6 (14.64%)	
Noncardiac etiology	No Hospitalization	109 (79.56%)	50 (64.10%)	9 (21.95%)	<0.001
	Pneumonia, RF	17 (12.41%)	21 (26.92%)	25(60.98%)	
	Other	11 (8.03%)	7 (8.97%)	7 (17.07%)	
A patient who has received at least two doses of the vaccıne		89 (64.96%)	50 (64.10%)	24 (58.54%)	0.756
Inactivated vaccines		53 (38.69%)	33 (42.31%)	20 (48.78%)	0.509
mRNA-based vaccine		60 (43.80%)	29 (37.18%)	8 (19.51%)	0.019
Tamponade		1 (0.07%)	3(3.84%)	5 (12.19%)	0.001
Cardiac Mortality.		3 (2.19%)	6 (7.69%)	9 (21.95%)	<0.001
Noncardiac Mortality		5 (3.65%)	7 (8.97%)	7 (17.07%)	0.014
Total Mortality		8 (5.84%)	13 (16.67%)	16 (39.02%)	<0.001
Symptoms in Outpatient Clinic Controls	No Symptoms	33 (24.09%)	19 (24.68%)	0 (0.00%)	NA
	Palpitation	18 (13.14%)	6 (7.79%)	4 (10.26%)	
	Dyspnea	30 (21.90%)	30 (38.96%)	29 (74.36%)	
	Chest Pain	38 (27.74%)	21 (27.27%)	6 (15.38%)	
	Other	18 (13.14%)	1 (1.30%)	0 (0.00%)	
Association of at least two doses of vaccination and mortality in patients who survived and died during the total follow-up					
	Alive: 219		Death: 37	*p* Value	
Inactivated vaccines/n (%)	99 (45.2%)		7 (18.9%)	0.003	
mRNA-based vaccine/n (%)	94 (42.9%)		3 (8.1%)	0.000	

CT, computerized tomography; PE, pericardial effusion; HF, heart failure; AF, atrial fibrillation; ACS, acute coronary syndrome; RF, respiratory failure; N/A, Not available.

## Data Availability

The data that support the findings of this study are available from the corresponding author upon reasonable request.
